# Sexual transmission of urogenital bacteria: whole metagenome sequencing evidence from a sexual network study

**DOI:** 10.1128/msphere.00030-24

**Published:** 2024-02-15

**Authors:** Kayla A. Carter, Michael T. France, Lindsay Rutt, Lisa Bilski, Sebastian Martinez-Greiwe, Mary Regan, Rebecca M. Brotman, Jacques Ravel

**Affiliations:** 1 Institute for Genome Sciences, University of Maryland School of Medicine, Baltimore, Maryland, USA; 2 Department of Microbiology and Immunology, University of Maryland School of Medicine, Baltimore, Maryland, USA; 3 School of Nursing, University of Maryland, Baltimore, Maryland, USA; 4 Department of Epidemiology and Public Health, University of Maryland School of Medicine, Baltimore, Maryland, USA; University of California, Davis, Davis, California, USA

**Keywords:** urogenital microbiome, vaginal microbiome, penile microbiome, bacterial vaginosis, metagenomics, strain concordance

## Abstract

Sexual transmission of the urogenital microbiota may contribute to adverse sexual and reproductive health outcomes. The extent of sexual transmission of the urogenital microbiota is unclear as prior studies largely investigated specific pathogens. We used epidemiologic data and whole metagenome sequencing to characterize urogenital microbiota strain concordance between participants of a sexual network study. Individuals who screened positive for genital *Chlamydia trachomatis* were enrolled and referred their sexual contacts from the prior 60–180 days. Snowball recruitment of sexual contacts continued for up to four waves. Vaginal swabs and penile urethral swabs were collected for whole metagenome sequencing. We evaluated bacterial strain concordance using inStrain and network analysis. We defined concordance as ≥99.99% average nucleotide identity over ≥50% shared coverage; we defined putative sexual transmission as concordance between sexual contacts with <5 single-nucleotide polymorphisms per megabase. Of 138 participants, 74 (54%) were female; 120 (87%) had genital chlamydia; and 43 (31%) were recruited contacts. We identified 115 strain-concordance events among 54 participants representing 25 bacterial species. Seven events (6%) were between sexual contacts including putative heterosexual transmission of *Fannyhessea vaginae*, *Gardnerella leopoldii*, *Prevotella amnii*, *Sneathia sanguinegens*, and *Sneathia vaginalis* (one strain each), and putative sexual transmission of *Lactobacillus iners* between female contacts. Most concordance events (108, 94%) were between non-contacts, including eight female participants connected through 18 *Lactobacillus crispatus* and 3 *Lactobacillus jensenii* concordant strains, and 14 female and 2 male participants densely interconnected through 52 *Gardnerella swidsinskii* concordance events.

**IMPORTANCE:**

Epidemiologic evidence consistently indicates bacterial vaginosis (BV) is sexually associated and may be sexually transmitted, though sexual transmission remains subject to debate. This study is not capable of demonstrating BV sexual transmission; however, we do provide strain-level metagenomic evidence that strongly supports heterosexual transmission of BV-associated species. These findings strengthen the evidence base that supports ongoing investigations of concurrent male partner treatment for reducing BV recurrence. Our data suggest that measuring the impact of male partner treatment on *F. vaginae*, *G. leopoldii*, *P. amnii*, *S. sanguinegens*, and *S. vaginalis* may provide insight into why a regimen does or does not perform well. We also observed a high degree of strain concordance between non-sexual-contact female participants. We posit that this may reflect limited dispersal capacity of vaginal bacteria coupled with individuals’ comembership in regional transmission networks where transmission may occur between parent and child at birth, cohabiting individuals, or sexual contacts.

## INTRODUCTION

The urogenital microbiome is a primary line of defense against sexually transmitted infections (STI), including HIV (1). The vaginal microbiome of reproductive-age cisgender women is often dominated by a single *Lactobacillus* species (2). Dominance of D-lactic acid (LA) producing lactobacilli, including *Lactobacillus crispatus*, *Lactobacillus gasseri*, and *Lactobacillus jensenii*, is considered optimal due to strong and consistent associations with positive sexual and reproductive health outcomes (3, 4) and D-LA’s beneficial effects on host epithelium (5, 6). *Lactobacillus iners*, which is believed to be the most prevalent and abundant vaginal bacterial species worldwide (2, 7–9), produces only L-LA (6, 10). Compared to D-LA-producing lactobacilli, *L. iners* dominance appears to provide less protection against STI and is approximately twice as likely to shift toward diverse, non-optimal communities resembling bacterial vaginosis (BV) (6–9, 11, 12). BV is characterized by low/no lactobacilli and abundant Gram-negative anaerobes, including *Gardnerella*, “*Candidatus* Lachnocurva vaginae” (formerly BV-associated bacterium 1 [13]), *Fannyhessea* (formerly *Atopobium* [14]), *Prevotella*, and *Sneathia* (15–17). A quarter to third of women have BV; around half of cases are symptomatic with thin, gray/white, homogeneous vaginal discharge and/or fishy/amine vaginal odor (15, 17). Symptoms can severely negatively impact psychosocial well-being, self-esteem, and sexual satisfaction (18), and BV is associated with various adverse outcomes including HIV/STI acquisition, preterm birth, and cervical disease progression (19–22). First-line BV treatments include metronidazole and clindamycin; however, recurrence is high with as many as 60% having a recurrent episode within 1 year (23).

The penile microbiome is less well studied than the vaginal microbiome, but evidence suggests it may play a role in modulating local inflammation and HIV risk (1, 24). This study characterized the penile urethral microbiome, so we focus on the penile urethral microbiome here, as opposed to the penile cutaneous microbiome. Single-species dominance is less common in the male urethra than the vagina (25–29), and the overall abundance of penile bacteria is typically lower than of vaginal bacteria (25, 28). *Corynebacterium*, *Finegoldia*, *Peptoniphilus*, *Staphylococcus*, and *Streptococcus* are commonly detected in the male urethra (25–29). Their abundance differs by circumcision status, with *Corynebacterium*, *Staphylococcus*, and *Streptococcus* being higher among circumcised individuals and *Finegoldia* and *Peptoniphilus* higher among individuals with intact foreskin (27, 29). Vaginal lactobacilli, namely, *L. iners*, and BV-associated bacteria, primarily *Gardnerella*, *Prevotella*, and *Sneathia*, are also regularly detected in the male urethra (25–29). Vaginal bacteria are more prevalent and abundant in urethras of men reporting vaginal sex and men whose sex partners have BV (25–27, 29), and penile urethral microbiota composition can robustly predict incident BV in sex partners (27). In the vagina and male urethra, BV-associated anaerobes are linked to increased local levels of pro-inflammatory immune mediators (1, 24, 30).

Detecting vaginal bacteria in the penile microbiome suggests they may be sexually transmitted. Likewise, epidemiologic evidence consistently indicates BV may be sexually transmitted (between heterosexual contacts and between women who have sex with women [WSW]) (31–34), although this is debated (35–38). Early trials of BV male partner treatment (concurrently treating male sex partners of women with BV using similar regimens) showed little benefit for reducing BV recurrence; however, these trials had serious methodologic limitations and cannot be taken as strong evidence against BV sexual transmission (39–46). There is renewed interest in BV male partner treatment (28, 47–49), and while ongoing work addresses major limitations of early trials, the design and evaluation of BV male partner treatment regimens are constrained by our limited understanding of sexual transmission of urogenital bacteria. Prior molecular biology studies of strain-level urogenital bacterial concordance are restricted to a prespecified genus or species, predominantly overt pathogens (50–56). Prior metataxonomic studies of species-level concordance do not provide sufficient resolution to infer transmission (26, 28, 49, 57, 58). To address these limitations, we used genome-resolved metagenomics to identify concordant strains in the vaginal and penile microbiomes of individuals enrolled in the Sexually Transmitted Infection Network Groups (STING) study.

## RESULTS

**Fig 1:**
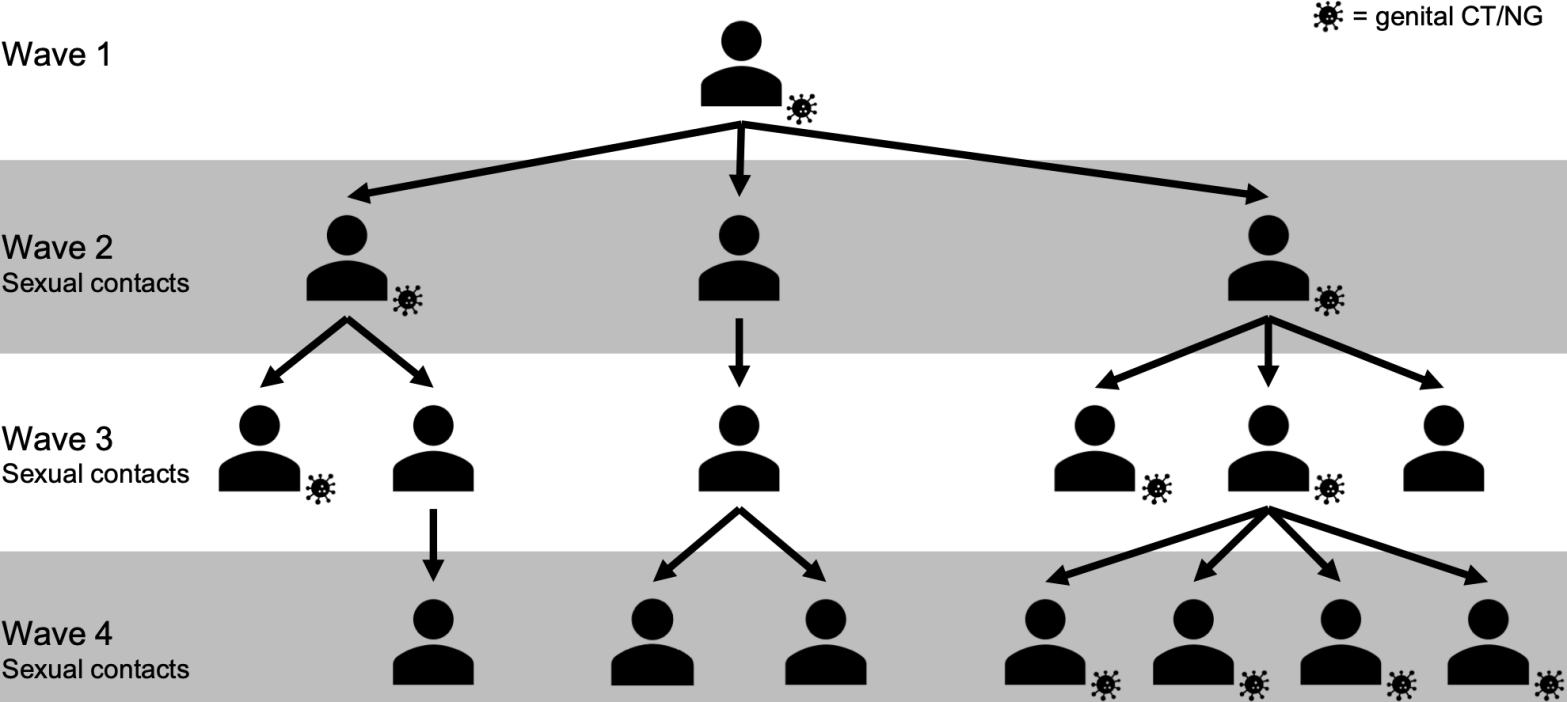
Hypothetical example of STING snowball recruitment. Wave 1 participants who tested positive for genital CT referred their sexual contacts from the prior 60 or 180 days (switched from 60 to 180 partway through recruitment) for enrollment in wave 2. Snowball recruitment of sexual contacts continued for up to four waves. Individuals with genital CT infection are indicated by the cartoon bacterium on their bottom right. This is a hypothetical example; it does not depict enrollment patterns observed in STING.

Participants were recruited from seven clinics providing genital *Chlamydia trachomatis* (CT) and *Neisseria gonorrhoeae* (NG) screening in the Baltimore, MD, USA, metropolitan area: four clinics serving the general population and three pop-up clinics serving large universities. Female and male individuals were eligible if they screened positive for genital CT by nucleic acid amplification test (NAAT), were 16–40 years old and were willing to provide names and contact information of all sexual contacts from the prior 60 or 180 days (switched from 60 to 180 partway through recruitment). These participants will be referred to as wave 1. Sexual contacts of wave 1 participants were then invited to enroll. Contacts were eligible if they were 16–40 years old, willing to undergo CT/NG screening, and willing to provide names and contact information of sexual contacts from the prior 60 or 180 days. These participants will be referred to as wave 2. Recruiting sexual contacts via this snowball approach continued for up to four waves (Fig. 1).

All participants underwent a reproductive tract examination, during which vaginal or penile urethral swabs were collected. Swabs were used for whole metagenome sequencing, and metagenomes were successfully sequenced for 138 of 139 participants (median 48,744,779 read pairs; range: 132,491–179,383,783). Of these 138 participants, half were female, two-thirds reported Black race, and the median age was 21 (Table 1). Most participants (87%) had CT; two had NG. Ninety-three participants were enrolled in wave 1, 63 of which (46% of total) had no sexual contacts enrolled. The remaining 33 wave 1 participants (24%) had 40 sexual contacts enrolled (wave 2, 29%). Wave 2 participants had two sexual contacts enrolled (wave 3), and wave 3 participants had one sexual contact enrolled (wave 4).

**TABLE 1:** Participant characteristics overall and stratified by gender

Characteristics	All participants (*N* = 138)		Female participants (*n* = 74)		Male participants (*n* = 64)	
	*n*	%	*n*	%	*n*	%
Race						
Asian	5	4	4	5	1	2
Black	92	67	49	66	43	67
Hispanic	5	4	2	3	3	5
Mixed	6	4	4	5	2	3
white	30	22	15	20	15	23
Age (years)^*a*^	21	19–23	20	19–22	21	20–24
Lifetime male sexual contacts^*a*^	2.5	0–9	8	3–10	0	0–0
Prefer not to answer	8	6	3	4	5	8
Lifetime female sexual contacts^*a*^	1	0–8.25	0	0–0.5	9	4–20
Prefer not to answer	18	13	7	9	11	17
Current contraception use^*b*^						
Condom	–	–	38	51	–	–
Hormonal	–	–	41	55	–	–
Copper IUD	–	–	1	1	–	–
Other	–	–	16	22	–	–
Waves and sexual contacts^*c*^						
Wave 1, no contacts	63	46	38	51	25	39
Wave 1, index contact	33	24	25	34	8	13
Wave 2, sexual contact	40	29	10	14	30	47
Wave 3, sexual contact	2	1	1	1	1	2
Wave 4, sexual contact	1	1	0	0	1	2
Enrollment site^*d*^						
General population clinic	31	23	19	26	12	19
University pop-up clinic	65	47	44	59	21	33
Sexual contact^*d*^	42	30	11	15	31	48
CST^*e*^						
I, *L. crispatus* dominated	7	5	7	10	0	0
III, *L. iners* dominated	14	10	13	18	1	2
IV-A, high “*Ca*. Lachnocurva vaginae,” moderate *Gardnerella*	15	11	13	18	2	3
IV-B, high *Gardnerella*	63	46	34	46	29	45
IV-C, diverse, more even	35	25	4	5	31	48
V, *L. jensenii* dominated	4	3	3	4	1	2
Self-reported urogenital symptoms	50	36	40	54	10	16
Genital CT (NAAT)	120	87	71	96	49	77
Genital NG (NAAT)	2	1	0	0	2	3
Clinician-evaluated conditions^*f*^						
PID^*g*^	–	–	0	0	–	–
Abnormal vaginal discharge	–	–	11	15	–	–
Cervical discharge	–	–	11	15	–	–
Amsel BV	–	–	7	10	–	–
Mucopurulent cervicitis	–	–	1	1	–	–
Vulvovaginal candidiasis	–	–	5	7	–	–
Penile discharge	–	–	–	–	0	0
Urethritis	–	–	–	–	2	3
Circumcised	–	–	–	–	53	83
Not recorded	–	–	–	–	4	6

^*a*^ Continuous data are presented as median and interquartile range.

^*b*^ Only female participants were asked about current contraceptive use, so these data are only presented for female participants.

^*c*^ Wave and partnership counts overall and for male participants sum to 1 greater than the total because one male participant was included in waves 2 and 3. Percentages for overall and male participants are out of the total number of unique participants (i.e*.*, 138 overall and 64 males).

^*d*^ Enrollment site was recorded only for wave 1 participants, not for sexual contacts.

^*e*^ CSTs were assigned using VALENCIA; CST, community state type.

^*f*^ PID, vaginal and cervical discharge, mucopurulent cervicitis, and vulvovaginal candidiasis were evaluated only among female participants. Penile discharge, urethritis, and circumcision status were evaluated only among male participants.

^*g*^ PID, pelvic inflammatory disease.

### Genome-resolved metagenomics

We determined metagenome taxonomic composition by mapping to the VIRGO non-redundant gene catalog (59) and assigned community state types (CSTs) using VALENCIA (2). We performed *de novo* assembly on each metagenome, individually, using metaSPAdes (60) and divided assembled contigs into metagenome-assembled genomes (MAGs) using MaxBin2, MetaBAT2, and MetaDecoder (61–63). We consolidated and refined bins using MetaWRAP and CheckM (64, 65), retaining MAGs that were ≥90% complete and <5% contaminated for analyses. We retained 507 MAGs representing 97 taxa (Table S1); their median completeness and contamination were 98.1% (interquartile range ([IQR] 95.6%–99.4%) and 0.2% (IQR 0%-0.7%), respectively. MAG taxonomy was assigned using GTDB-Tk (66).

**Fig 2:**
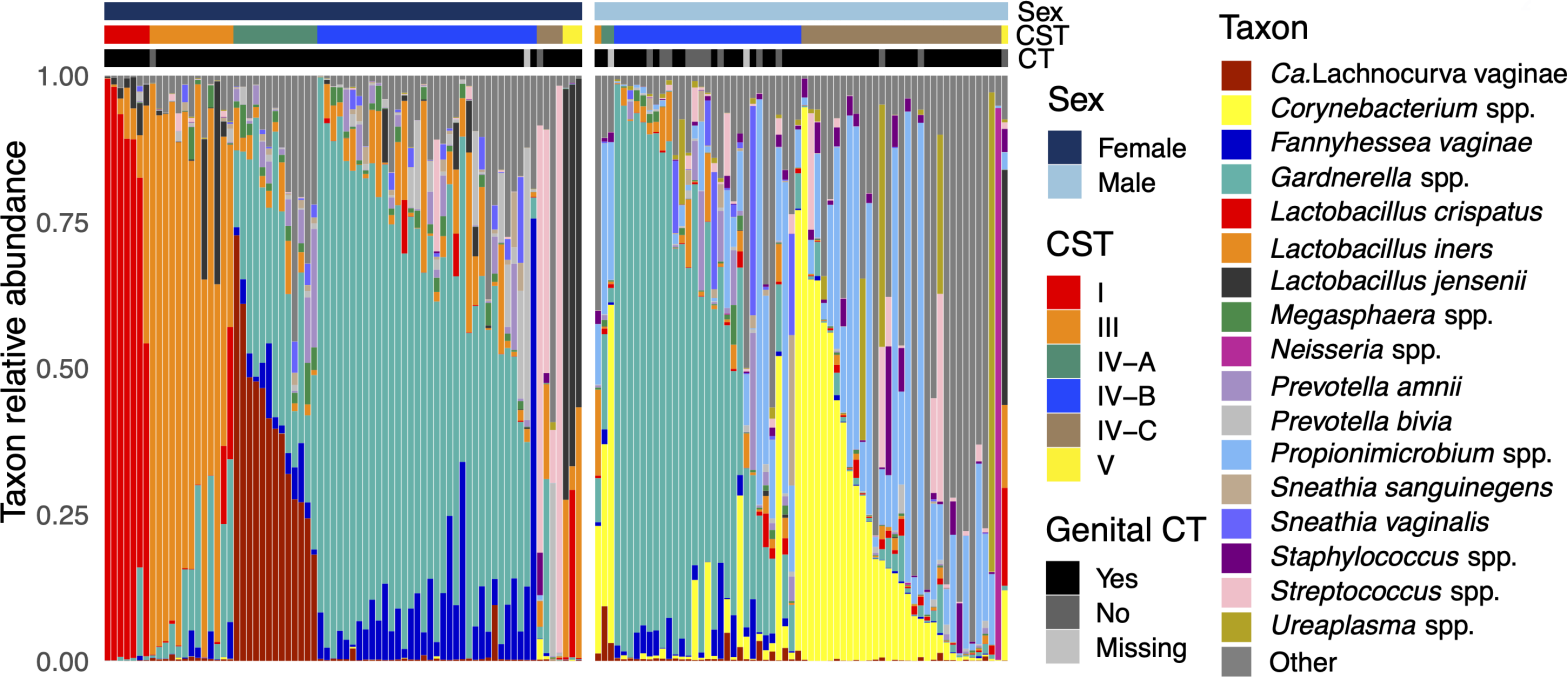
Urogenital microbiota composition, sex, and genital CT status of STING participants. Urogenital microbiota composition from whole metagenome sequencing. CSTs assigned using VALENCIA. The plot depicts the taxa with the top 10 mean relative abundances among female and male participants (calculated separately) as well as any remaining taxa that have a putative sexual transmission event.

While CST IV-B (high *Gardnerella* spp.) was prevalent among female and male participants (46% and 43%, respectively), most remaining female participants were assigned to CST I (*L. crispatus* dominated, 10%), CST III (*L. iners* dominated, 18%), or CST IV-A (high “*Ca*. L. vaginae,” 18%) (Fig. 2). Most remaining male participants were assigned to CST IV-C (moderate-to-high *Corynebacterium* spp., *Propionimicrobium* spp., 48%).

**Fig 3:**
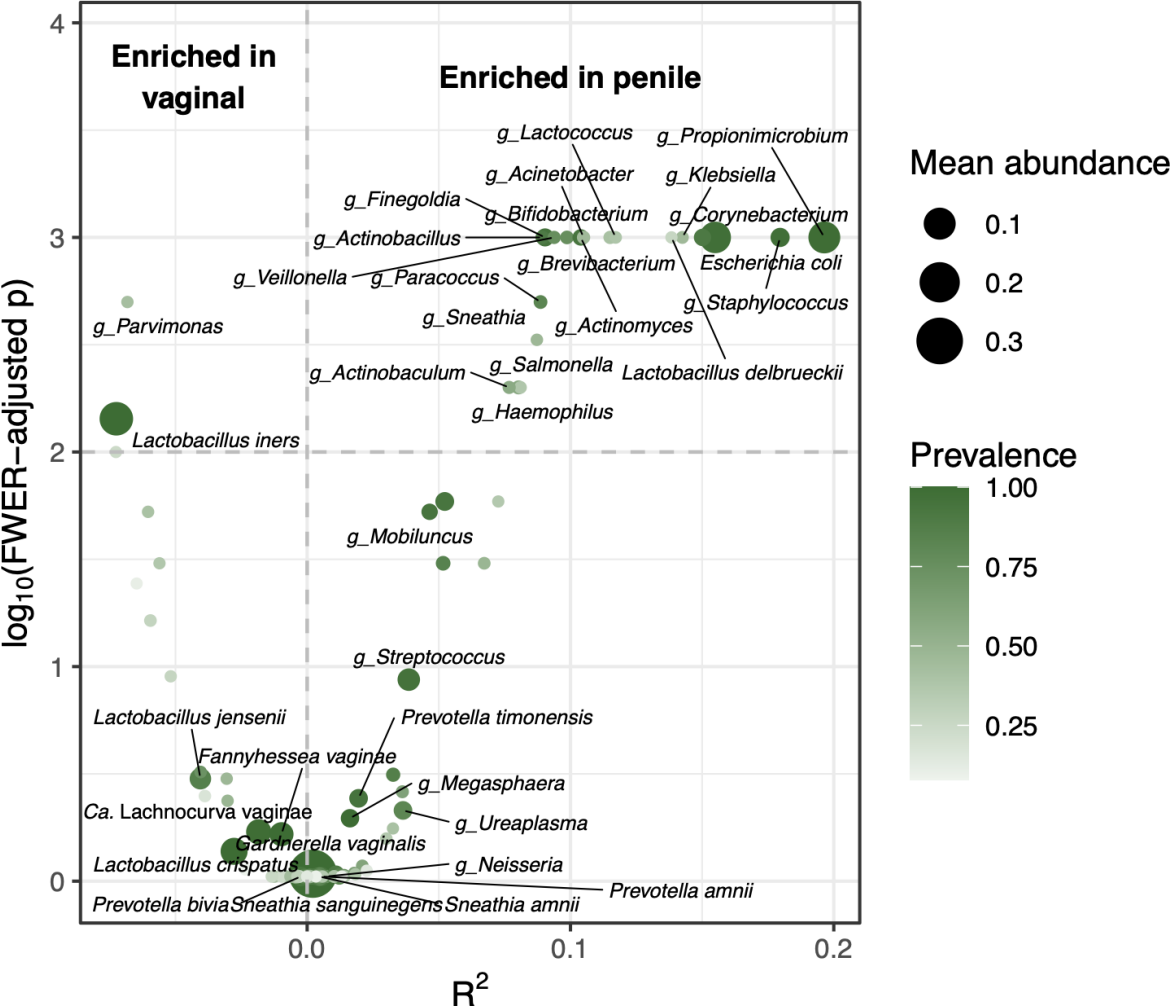
Differential abundance of taxa between vaginal and penile metagenomes. *R*^2^ is the proportion of variance in metagenome composition (across vaginal and penile samples) explained by a given taxon. The vertical dashed line at *R*^2^ = 0 indicates no difference in abundance between vaginal and penile metagenomes, negative *R*^2^ values indicate enrichment in vaginal metagenomes, and positive *R*^2^ values indicate enrichment in penile metagenomes. We controlled the FWER at 1%, as indicated by the horizontal dashed line at log_10_(FWER − adjusted *P*) = 2. All taxa that are differentially abundant at this FWER fall above the horizontal line and are labeled. Taxa that are presented in other figures are also labeled, regardless of significance.

We used ZicoSeq to compare vaginal and penile metagenome relative abundances (67). Controlling family-wise error rate (FWER) at 1%, we found that *L. iners* and *Parvimonas* spp. were significantly enriched in vaginal metagenomes compared to penile (Fig. 3). Nineteen taxa were significantly enriched in penile metagenomes compared to vaginal: *Acinetobacter* spp., *Actinobacillus* spp., *Actinobaculum* spp., *Actinomyces* spp., *Bifidobacterium* spp., *Brevibacterium* spp., *Corynebacterium* spp., *Escherichia coli*, *Finegoldia* spp., *Haemophilus* spp., *Klebsiella* spp., *Lactobacillus delbrueckii*, *Lactococcus* spp., *Paracoccus* spp., *Propionimicrobium* spp., *Salmonella* spp., *Sneathia* spp., *Staphylococcus* spp., and *Veillonella* spp. We additionally compared abundances according to CT status among penile metagenomes, which showed minor differences (Fig. S1). We did not perform a similar analysis among vaginal metagenomes as all but three female participants had CT.

### Strain-concordance summary

We used inStrain to identify concordant strains between participants (68). We used an all-vs-all strategy to build inStrain profiles for each metagenome-MAG pair by mapping sequence reads against MAGs using Bowtie2 (69). To identify strain concordance, we applied stringent filters requiring ≥99.99% sequence similarity [population average nucleotide identity (popANI)] over ≥50% shared coverage.

**Fig 4:**
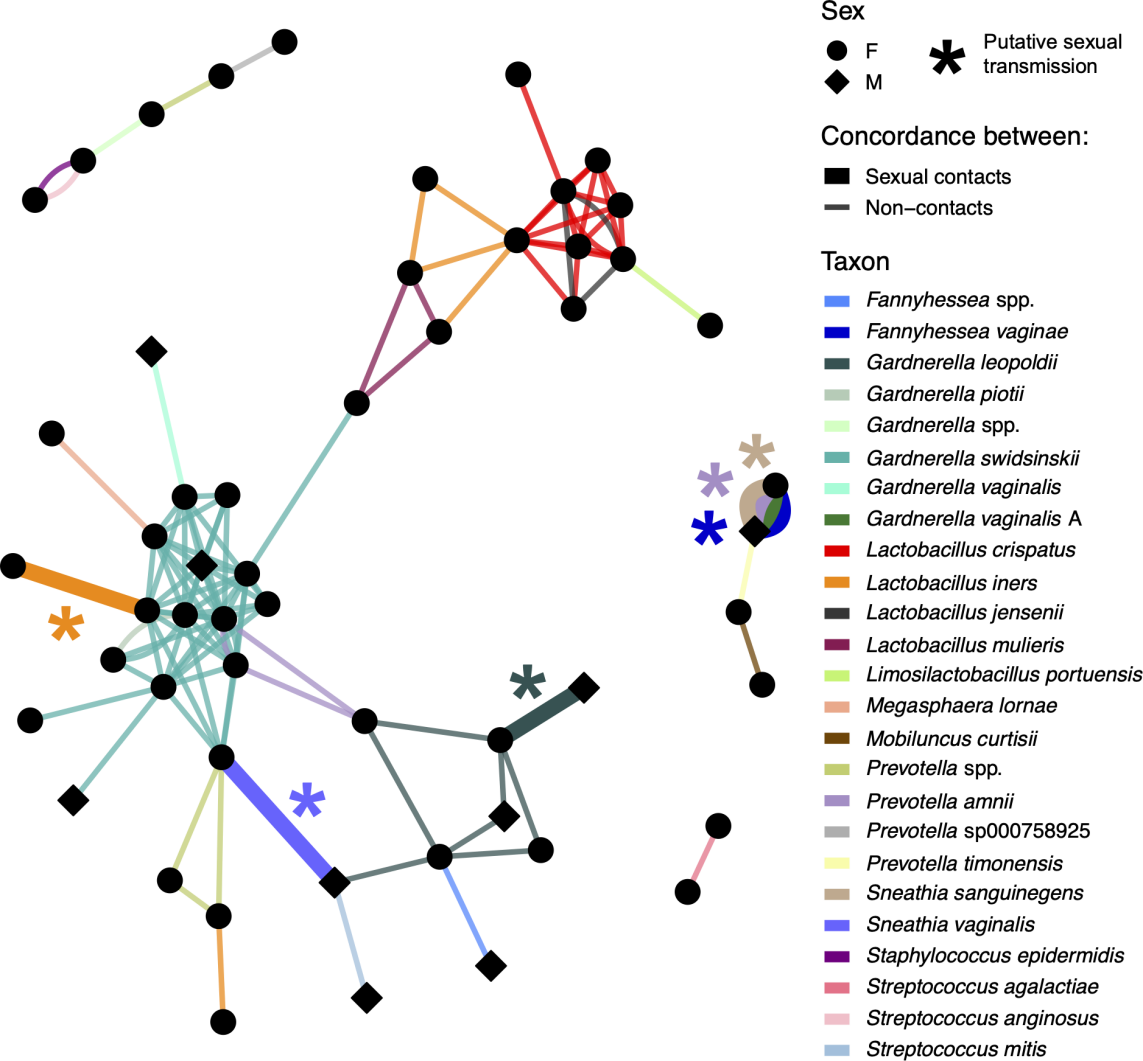
Network diagram of strain-concordance events between sexual contacts and non-contacts. Each node (circle or diamond) represents one participant. Each edge (line between nodes) represents a strain-concordance event between connected nodes. Thin edges indicate concordance between non-contacts. Thick edges indicate concordance between sexual contacts. Putative sexual transmission is indicated by colored asterisks adjacent to and colored the same as edges representing sexual transmission. Concordant strains were required to have ≥99.99% popANI across ≥50% of shared coverage. Sexual transmission was defined as strain concordance between sexual contacts with <5 popSNP/Mbp. popANI is estimated by inStrain based on popSNPs, which are called when two samples share no alleles at a given site. F, female; M, male.

**Fig 5:**
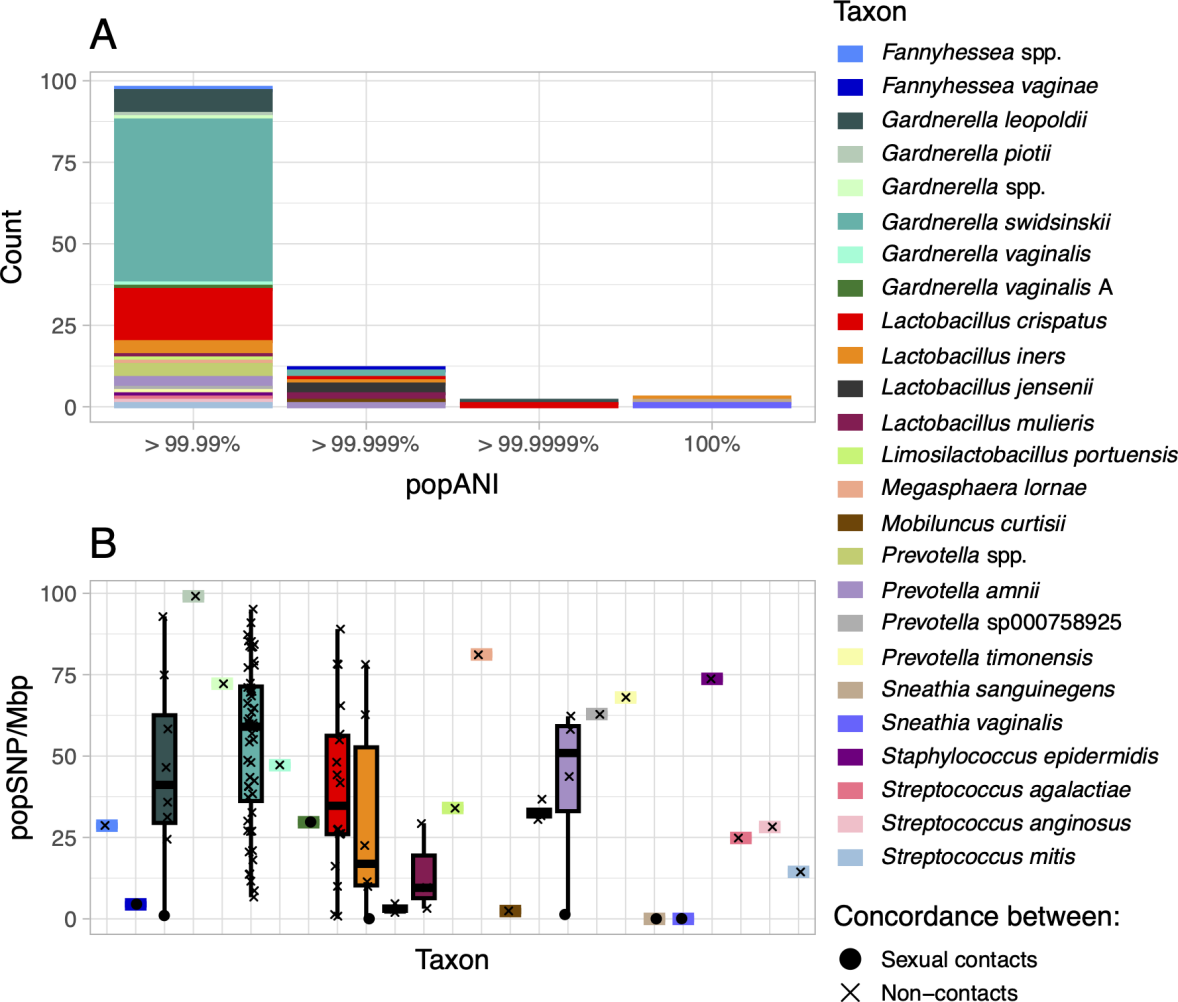
Relatedness between concordant strains. Histogram of popANI between concordant strains, colored by taxon (**A**) Dot, box, and whisker plots of popSNP/Mbp between concordant strains, colored by taxon. (**B**) inStrain defines popANI based on popSNPs, which are called when two samples share no alleles at a given site.

We identified 115 concordant strains among 54 participants representing 25 taxa (Fig. 4). For 17 taxa (68%), we observed one concordance event (Tables 2 and 3). The remaining concordant taxa were *Gardnerella leopoldii* (*n* = 8 events), *Gardnerella swidsinskii* (*n* = 52), *L. crispatus* (*n* = 18), *L. iners* (*n* = 6), *L. jensenii* (*n* = 3), *Lactobacillus mulieris* (formerly *L. jensenii* [70]) (*n* = 3), *Prevotella* spp. (*n* = 4), and *Prevotella amnii* (*n* = 4). Several taxa were concordant exclusively between sexual contacts (*Fannyhessea vaginae*, *Gardnerella vaginalis* A, *Sneathia sanguinegens*, *Sneathia vaginalis* ([formerly *Sneathia amnii*] [71]); some were concordant between contacts and non-contacts (*G. leopoldii*, *L. iners*, and *P. amnii*), and the remaining 18 were exclusively concordant between non-contacts. Relative abundances of concordant strains generally reflected their relative abundances in the study overall (Fig. 2; Tables 2 and 3). The distribution of population single-nucleotide polymorphisms (popSNP) per megabase (Mbp) differed across taxa (lowest median = 3 for *L. jensenii*, highest median = 59 for *G. swidsinskii*; Kruskal-Wallis *P* = 0.02; Fig. 5). There were fewer popSNP/Mbp when strains were concordant between sexual contacts (contacts median = 1, non-contacts median = 47; Kruskal-Wallis *P* = 9×10^−5^).

**TABLE 2:** Interpersonal characteristics of strain-concordance events between sexual contacts

Dyad members	Taxon	*N* concordance events within*^a^*:		Same CST^*b*^	Weeks between enrollment	Relative abundances in dyad	
		Heterosexual dyad	Woman-woman dyad			Minimum	Maximum
STING106 STING224	*Fannyhessea vaginae*	1	0	Yes	1	14	16
	*Gardnerella vaginalis* A^*c*^	1	0	Yes	1	38	51
	*Prevotella amnii*	1	0	Yes	1	9	15
	*Sneathia sanguinegens*	1	0	Yes	1	4	9
STING154 STING190	*Gardnerella leopoldii^c^*	1	0	Yes	0	58	88
STING168 STING201	*Sneathia vaginalis*	1	0	Yes	0	3	36
STING117 STING118	*Lactobacillus iners*	0	1	No	0.3	15	63

^*a*^ Strain concordance defined as ≥99.99% popANI over ≥50% of the length of the genomes. inStrain defines popANI based on popSNPs, which are called when two samples share no alleles at a given site.

^*b*^ CSTs assigned using VALENCIA.

^*c*^ For *G. vaginalis* A and *G. leopoldii* concordance events, relative abundances are presented for *Gardnerella* spp. at the genus level.

**TABLE 3:** Interpersonal characteristics of strain-concordance events between non-contacts

Taxon^*a*^	*N* concordance events^*b*^	Concordant between:										Relative abundances in non-contact pairs with concordance		Relative abundance ratio between non-contacts with concordance	
		Female participants		Female and male participants		Male participants		Same CST^*c*^		Weeks between enrollment						
		*N*	Row %	*N*	Row %	*N*	Row %	*N*	Row %	Med^*d*^	IQR^*d*^	Minimum	Maximum	Med^*d*^	Range
*Fannyhessea* spp.	1	0	0	1	100	0	0	1	100	63	–	4	5	1.1	–
*Gardnerella leopoldii*	7	4	57	3	43	0	0	5	71	54	24–96	13	88	1.7	1.1–6.7
*Gardnerella piotii*	1	1	100	0	0	0	0	1	100	4	**–**	56	75	1.3	–
*Gardnerella* spp.	1	1	100	0	0	0	0	0	0	149	–	29	30	1.0	–
*Gardnerella swidsinskii*	52	42	81	10	19	0	0	30	58	77	32–111	30	96	1.4	1.0–3.2
*Gardnerella vaginalis*	1	0	0	1	100	0	0	1	100	2	–	78	85	1.1	–
*Lactobacillus crispatus*	18	18	100	0	0	0	0	16	89	5	2–154	29	99	1.1	1.0–3.5
*Lactobacillus iners*	5	5	100	0	0	0	0	0	0	50	29–79	6	61	4.9	1.6–7.7
*Lactobacillus jensenii*	3	3	100	0	0	0	0	1	33	27	15–28	2	65	12.2	3.0–36.5
*Lactobacillus mulieris*	3	3	100	0	0	0	0	0	0	93	48–94	8	56	4.3	1.6–7.0
*Limosilactobacillus portuensis*	1	1	100	0	0	0	0	0	0	34	–	0.2	1	2.7	–
*Megasphaera lornae*	1	1	100	0	0	0	0	1	100	78	–	2	7	3.8	–
*Mobiluncus curtisii*	1	1	100	0	0	0	0	1	100	38	--	2	3	1.7	--
*Prevotella* spp.	4	4	100	0	0	0	0	2	50	48	36–53	–	–	–	–
*Prevotella amnii*	3	3	100	0	0	0	0	1	33	66	34–67	2	7	2.1	1.6–3.4
*Prevotella* sp000758925	1	1	100	0	0	0	0	0	0	22	–	–	–	–	–
*Prevotella timonensis*	1	0	0	1	100	0	0	1	100	184	–	3	9	3.0	–
*Staphylococcus epidermidis*	1	1	100	0	0	0	0	1	100	197	–	2	7	4.4	–
*Streptococcus agalactiae*	1	1	100	0	0	0	0	0	0	190	–	19	97	5.1	–
*Streptococcus anginosus*	1	1	100	0	0	0	0	1	100	197	–	42	73	4.4	–
*Streptococcus mitis*	1	0	0	0	0	1	100	0	0	53	–	0.4	36	91.3	–

^*a*^ Relative abundance data are not presented for *Prevotella* spp. or *Prevotella* sp000758925 because there was no clear comparator taxon. Genus-level relative abundance data are presented for *Fannyhessea* spp., all *Gardnerella* spp., *Megasphaera lornae*, *Mobiluncus curtisii*, *Staphylococcus epidermidis*, and all *Streptococcus* spp. For *Lactobacillus mulieris*, relative abundance data from *Lactobacillus jensenii* are presented due to reclassification. For *Limosilactobacillus portuensis*, relative abundance data from *Lactobacillus vaginalis* are presented due to reclassification.

^*b*^ Strain concordance defined as ≥0.9999 popANI over ≥50% of the length of the genomes. inStrain defines popANI based on popSNPs, which are called when two samples share no alleles at a given site.

^*c*^ CSTs assigned using VALENCIA.

^*d*^ IQR, interquartile range; Med, median.

### Strain concordance between sexual contacts

Seven concordance events (6%) were between sexual contacts in four dyads. Strain concordance between contacts does not necessarily reflect sexual transmission within the dyad, so we applied additional criteria to determine which events likely represent recent sexual transmission. We considered strains that were concordant between contacts with <5 popSNP/Mbp to be sexually transmitted. Of seven concordance events between contacts, six met this criterion.

**Fig 6:**
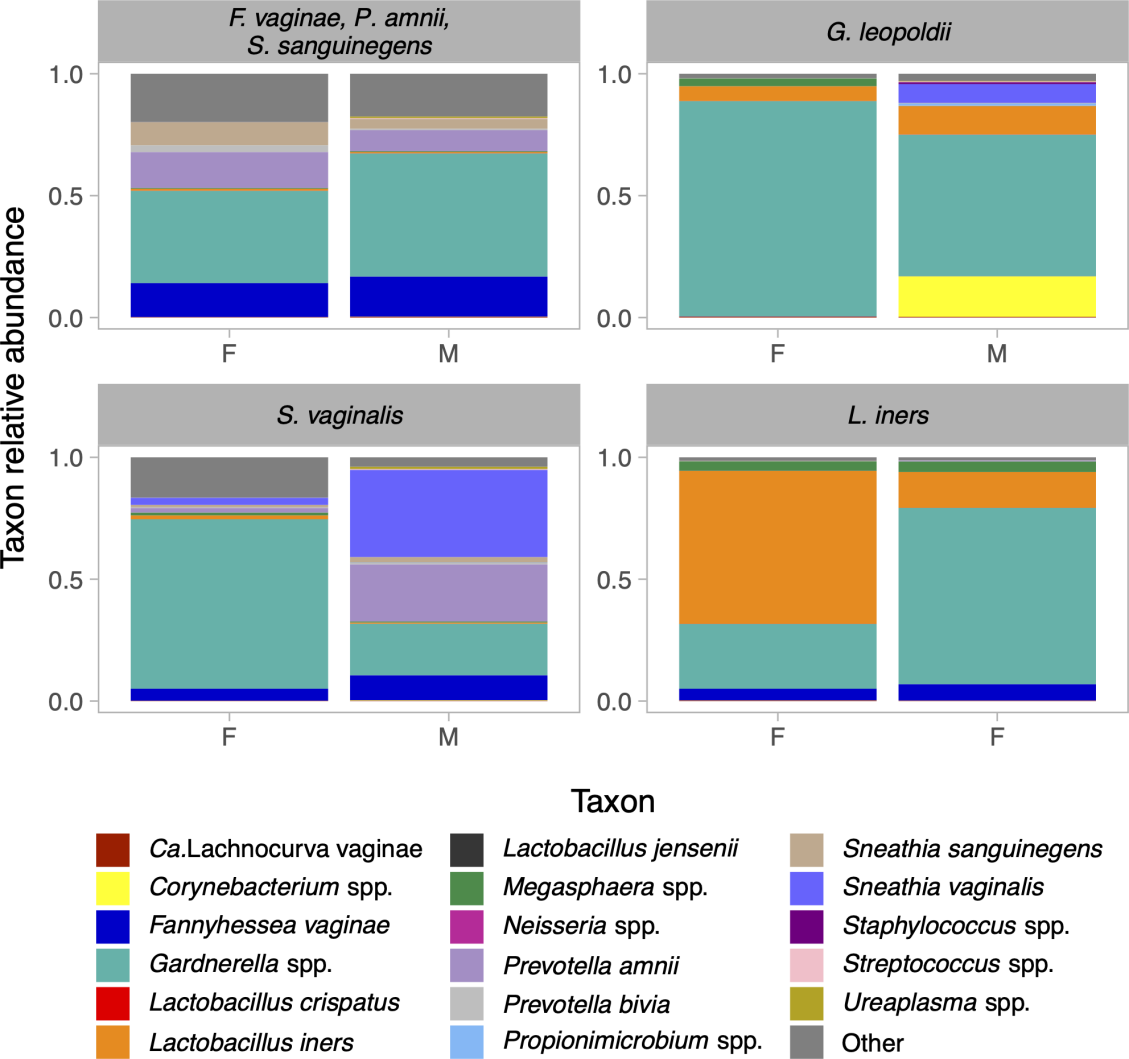
Urogenital microbiota composition of sexual contacts with concordant strains. Each panel depicts urogenital microbiota composition for a unique sexual contact dyad with ≥1 concordant strains. The label at the top of each panel indicates the species of concordant strains that were identified as putatively sexually transmitted, and the *x*-axis labels indicate the sex of each contact.

Heterosexual contacts (STING106 and STING224) enrolled 7 days apart had concordant *F. vaginae* (4.5 popSNP/Mbp), *G. vaginalis* A (30 popSNP/Mbp), *P. amnii* (1.3 popSNP/Mbp), and *S. sanguinegens* (0 popSNP/Mbp; Fig. 4, right middle). We consider these *F. vaginae*, *P. amnii*, and *S. sanguinegens* strains to be heterosexually transmitted. Both contacts were assigned to CST IV-B (Fig. 6). The female contact had CT and reported current contraceptive implant, current bloody vaginal discharge, and no BV history. The male contact was circumcised, screened negative for CT, and reported no symptoms (clinician noted scant urethral discharge). Both contacts reported being regular partners. Their most recent sex was 7–14 days previously, and they had sex three to five times in the prior 60 days with no condom use.

Heterosexual contacts (STING154 and STING190) enrolled on the same day had concordant *G. leopoldii* (1.0 popSNP/Mbp; Fig. 4, bottom middle), which we consider heterosexually transmitted. Both contacts were assigned to CST IV-B and reported no symptoms. The female contact had CT and reported no BV history. The male contact was circumcised and screened negative for CT. Both contacts reported being occasional partners; their last sex was 1 month previously, and they had sex two to three times in the prior 60 days with no condom use.

Heterosexual contacts (STING168 and STING201) enrolled on the same day had concordant *S. vaginalis* (0 popSNP/Mbp; Fig. 4, bottom left), which we consider heterosexually transmitted. Both contacts were assigned to CST IV-B and had CT. The female contact reported current abnormal vaginal discharge (clinician reported no BV findings), current copper intrauterine device, and no BV history. The male contact was circumcised and reported no symptoms. These contacts reported discrepant characteristics of their sexual relationship. The female contact reported being regular partners; their last sex was 4 days previously, and they had sex four times in the prior 60 days with intermittent condom use. The male contact reported being new partners; their last sex was 0 days previously (within the prior 24 hours), and they had sex once with no condom use.

Female sexual contacts (STING117 and STING118) enrolled two days apart had concordant *L. iners* (0 popSNP/Mbp; Fig. 4, left middle), which we consider sexually transmitted. This was the only *Lactobacillus* strain concordant between contacts (no *Lactobacillus* concordance between heterosexual contacts). One contact each was assigned to CST III and CST IV-B. Both contacts had CT and reported no symptoms and no BV history; one contact reported current oral contraceptive use. They reported being regular partners; their last sex was 0 days prior to the first contact enrolling (within the prior 24 hours), and they had sex >20 times in the prior 60 days.

### Strain concordance between non-contacts

Most concordance events (108, 94%) were between non-contacts, including 91 (84% of non-contact concordance) between female participants, 16 (15%) between female and male participants, and 1 (1%) between male participants.

Eight female participants were interconnected through 18 *L*. *crispatus* and 3 *L. jensenii* concordant strains (Fig. 4 top right). Seventeen of these concordance events were between participants assigned to CST I; four were between participants assigned to CST I and CST V (Fig. S2). Median popSNP/Mbp between non-contacts was 35 for *L. crispatus* and 2.9 for *L. jensenii*; each *L. jensenii* concordance event had <5 popSNP/Mbp. Participants concordant for *L. crispatus* were enrolled a median of 5 weeks apart; those concordant for *L. jensenii* were enrolled a median of 27 weeks apart.

Fourteen female and two male participants were densely interconnected through 52 *G*. *swidsinskii* concordance events (Fig. 4 left middle). Twenty-eight of these events were between participants assigned to CST IV-B, 22 between participants assigned to CST IV-A and CST IV-B, and 2 between participants assigned to CST IV-A (Fig. S3). All *G. swidsinskii* concordant strains showed >5 popSNP/Mbp (median = 59). Participants concordant for *G. swidsinskii* were enrolled a median of 77 weeks apart.

### Comparing concordance between contacts and non-contacts

Because nearly half of participants did not have a contact enrolled and few concordance events were between contacts, we examined the prevalence of concordance/transmission among contacts and non-contacts. For non-contacts, we defined the prevalence denominator as the total number of unique participant pairs minus the number of contact dyads, which represents all possible opportunities for non-contact concordance. The aim here is to describe the relative frequency at which we observed concordance between contacts vs between non-contacts, not to generate precise population-level prevalence estimates.

With 138 participants, there are 9,453 unique 2-participant pairs. Forty-two were contact dyads, leaving 9,411 unique non-contact pairs. The prevalence of concordance/transmission was 9.5% between contacts (4 of 44 dyads had concordance/transmission) and 1.1% between non-contacts (104 of 9,411).

Considering putative heterosexual transmission, there were 74 female participants and 64 male, giving 4,736 unique female-male pairs. Thirty-nine were contact dyads, leaving 4,697 unique non-contact female-male pairs. The prevalence of concordance/transmission was 7.7% between female-male contact dyads (3 of 39) and 0.5% between non-contact female-male pairs (22 of 4,697).

Considering putative transmission between women, there were 2,701 unique female-female pairs. Two were contact dyads, leaving 2,699 unique non-contact female-female pairs. The prevalence of concordance/transmission was 50% between female-female contact dyads (1 of 2) and 3.2% between non-contact female-female pairs (87 of 2,699).

## DISCUSSION

We present strain-level metagenomic evidence that strongly supports heterosexual transmission of BV-associated bacteria and sexual transmission of *L. iners* between female contacts. These findings are consistent with prior molecular biology studies of strain-level urogenital bacterial concordance (50–56) and metataxonomic studies of species-level urogenital bacterial concordance (26, 28, 49, 57, 58) between sexual contacts. Importantly, genome-resolved metagenomics allowed us to address primary limitations of prior studies. Molecular biology studies characterized strain-level concordance but only for a prespecified genus or species (50–56). Because BV is a polymicrobial condition whose etiology remains unclear (3, 35, 36, 39), targeted approaches cannot examine strain concordance for all potentially relevant taxa. Metataxonomic studies used 16S rRNA gene amplicon sequencing to characterize concordance throughout the vaginal and penile microbiotas but are limited to the species level or higher (26, 28, 49, 57, 58). Because species exist as multi-strain communities in the vagina (10, 59, 72), transmission cannot be inferred from species-level concordance. Genome-resolved metagenomics instead provided us an untargeted approach to identify concordant strains (>99.99% popANI, <5 popSNP/Mbp) in the vaginal and penile microbiomes of recent sexual contacts, providing strong support for sexual transmission of several BV-associated species and *L. iners*.

Whether BV can be sexually transmitted is debated (35–38). This study cannot demonstrate heterosexual BV transmission; however, our observations do provide support for possible BV transmission. We identified putative heterosexual transmission of *F. vaginae*, *G. leopoldii*, *P. amnii*, *S. sanguinegens*, and *S. vaginalis*. Prior epidemiologic studies reported associations between these taxa and specific combinations of Amsel criteria; these associations were largely consistent between populations of Kenyan and American women (73, 74). *In vitro* and omics studies indicate these taxa may contribute to BV signs and symptoms via production of biogenic amines (75–77) or extracellular enzymes that degrade cervicovaginal mucus (78–80). Here, one of three female contacts with putative transmission of BV-associated taxa reported abnormal vaginal discharge (none reported odor). There were no clinical BV findings for these female contacts.

A primary argument against BV sexual transmission is that early male partner treatment trials showed little to no benefit for reducing BV recurrence (39–46). These trials are subject to major limitations including insufficient randomization methods, lack of power calculations, suboptimal BV treatment regimens for women, and lack of adherence data (39–46); that these trials did not demonstrate a reduction in BV recurrence among dyads randomized to male partner treatment should not be interpreted as evidence against heterosexual BV transmission. Recent years have seen a resurgence of interest in BV male partner treatment with multiple studies and trials completed and several under way (28, 47–49). Identifying putative heterosexual transmission of BV-associated taxa strengthens the evidence base supporting these ongoing investigations. While we cannot comment on potential transmission of additional BV-associated bacteria, measuring the impact of male partner treatment on *F. vaginae*, *G. leopoldii*, *P. amnii*, *S. sanguinegens*, and *S. vaginalis* may provide insight into a regimen’s performance. Two recent pilot studies evaluated a combined regimen of oral metronidazole and topical clindamycin for male partners, which showed reductions in penile *Prevotella* spp. (28, 49). No other taxa we observed to be heterosexually transmitted were significantly reduced.

Another major area of ongoing research aims to rigorously design and test *Lactobacillus* live biotherapeutic products (LBPs) to achieve/maintain optimal vaginal microbiota composition and vaginal health. Our observation of putative *L. iners* transmission between female contacts suggests *Lactobacillus* LBP may be particularly beneficial for WSW as the strain(s) may be transmitted within dyads and WSW sexual networks. Given high BV concordance between female sex partners (31, 32), transmission of beneficial lactobacilli through WSW sexual networks could yield substantial gains in individual and community health. That said, *Lactobacillus* LBP research largely focuses on *L. crispatus* (81, 82), which shows substantial genomic, metabolic, and epidemiologic differences to *L. iners* (8–10, 83, 84). Without identifying putative transmission of other lactobacilli, it is unclear how generalizable the observation of *L. iners* transmission is to possible transmission of *Lactobacillus* LBP strains.

While our data do not provide evidence of *L. crispatus* sexual transmission, they also cannot rule it out. Importantly, only 2 of 43 contact dyads included two women, limiting our insight into sexual transmission of vaginal bacteria between women. The minimum relative abundance of any putatively transmitted strain was 3% (*S. vaginalis*). *L. crispatus* relative abundances among female non-contacts with *L. crispatus* concordance ranged from 29% to 99%, with five of eight ≥89%. Conversely, *L. crispatus* relative abundances ranged from 0.02% to 0.10% among contacts in WSW dyads, and the maximum *L. crispatus* relative abundance among male participants in contact dyads was 17%, with most <1% (Fig. S4). These values suggest that if any contact dyad had sexually transmitted *L. crispatus*, our analyses likely are not sensitive enough to detect this concordance.

Finally, we observed frequent strain concordance between non-contacts, primarily *G. swidsinskii* and *L. crispatus* between female participants. Because concordance/transmission was 9–16 times more prevalent among contact dyads compared to non-contact pairs, strain concordance between non-contacts should not be taken as evidence against putative sexual transmission. Frequent strain concordance between female non-contacts is consistent with a prior analysis of vaginal bacterial strain concordance within and between families in the Baltimore area. While some reproductive-age mothers and their reproductive-age daughters had concordant strains, participants were more likely to have a concordant strain with someone they were not related to than someone in their family (85). Substantial strain concordance between female non-contacts is also consistent with a recent person-level meta-analysis of 7,646 stool and 2,069 saliva metagenomes from 20 countries that showed non-negligible strain concordance in these microbiomes between non-cohabitating individuals in the same community (86).

We hypothesize that a high degree of strain concordance between unrelated and/or non-sexual-contact women may reflect limited dispersal capacity of vaginal bacteria coupled with individuals’ comembership in regional transmission networks (85). Here, we are considering regions the size of a metropolitan area because STING recruited participants from the Baltimore metropolitan area. However, our hypotheses may apply to any area that encompasses a sexual and/or urogenital bacteria transmission network. Because vaginal lactobacilli and *Gardnerella* spp. are rarely detected in the environment or human body sites other than the genitals and many vaginal bacteria are anaerobic, we posit that direct person-to-person transmission (e.g., between parent and child at birth, cohabitating individuals, or sexual contacts) may represent a primary means of geographic dispersal of vaginal bacteria (85). Under this assumption, we would expect sexual network characteristics to influence vaginal bacterial dispersal. In sexual networks, small groups of high-contact individuals, termed core groups, play an integral role in maintaining bacterial STI endemicity/outbreaks (87). This can largely be attributed to assortative mixing and partner concurrency: core group members disproportionately partner with other core group members and are more likely to have concurrent partners than non-core-group members (87), which sustains STI transmission within core groups. When core group members partner with non-core-group members, this disseminates the STI in the general population. Similar processes may drive maintenance and dispersal of network-specific consortia of urogenital bacterial strains. Network characteristics appear to influence pathogen phylogenies (88); evaluating whether this is true for all or specific members of the vaginal microbiome could vastly improve our understanding of vaginal microbial ecology and biogeography as well as BV etiology. The sexual networks enrolled in STING were small and isolated (Fig. S5), so we were not able to test these hypotheses.

This study should be interpreted in the context of its limitations. While these metagenomic data provide strong evidence supporting sexual transmission of urogenital bacteria, the study design is insufficient to prove sexual transmission or identify the direction of putative transmission. We cannot determine whether putatively transmitted strains engrafted in “recipient” contacts’ urogenital microbiomes. It is possible strains failed to engraft, but sufficient DNA was present at sampling to detect the transient concordant strain.

Second, penile urethral swabs yielded significantly fewer reads than vaginal swabs (median non-host reads: 2.3 × 10^5^ for penile, 6.6 × 10^6^ for vaginal, Kruskal-Wallis *P* = 2 × 10^−16^; Fig. S6). For a given MAG and pair of metagenomes, inStrain only compares sites on the MAG with ≥5× coverage in both metagenomes. Per-MAG number of comparable sites (≥5× coverage) was significantly greater in vaginal metagenomes than penile (Kruskal-Wallis *P* < 2 × 10^−16^). Vaginal and penile metagenomes had similar proportions of metagenome-MAG mappings with 0 comparable sites (~54%); however, among metagenome-MAG mappings with >0 comparable sites, the median per-MAG number of comparable sites was 535 for vaginal metagenomes (IQR 190–1,895) and 302 for penile metagenomes (IQR 168–780). Having significantly fewer comparable sites in penile metagenomes likely prevented concordance events involving male participants from meeting our criteria of ≥99.99% popANI over >50% shared coverage, and our analyses likely under-detected strain concordance/transmission between male and female participants.

Finally, STING participants were enrolled in the Baltimore area, had high CT prevalence, and were relatively young. It is unclear how generalizable these findings are to other geographic regions, less-densely populated settings, older populations, or populations with lower STI burden.

By integrating genome-resolved metagenomics in a sexual network study, we provide insight into the sexual transmissibility of the urogenital microbiota and vaginal bacterial biogeography. Our findings may have substantial implications for achieving and maintaining vaginal health among sexually active individuals, particularly via novel interventions including BV partner treatment and LBP. We believe these data represent only “the tip of the iceberg” when it comes to urogenital bacterial transmission: we likely under-detected transmission in heterosexual and WSW dyads, and we cannot comment on how representative our findings are. Additional studies in various geographic contexts and sexual networks with various characteristics are warranted to broaden our understanding of sexual transmission of the urogenital microbiota, vaginal bacterial biogeography, and whether these vary across populations.

## MATERIALS AND METHODS

We used data and samples from the Sexually Transmitted Infection Network Groups study to evaluate urogenital bacterial strain concordance between sexual contacts and non-contacts. We report our results according to Strengthening the Organization and Reporting of Microbiome Studies guidelines (Table S2) (89).

### STING recruitment and procedures

Between February 2016 and March 2020, participants were recruited from seven clinics providing CT/NG screening in the Baltimore, MD, USA, metropolitan area. Individuals were eligible if they screened positive for genital CT by NAAT, were 16–40 years old, and were willing to provide the names and contact information of all sexual contacts from the prior 60 or 180 days. Individuals were ineligible if they were living with HIV, ever exchanged sex for money or drugs, were taking immunosuppressive medications, had a severe medical condition, or were non-English-speaking (all self-report). Female individuals were ineligible if they were pregnant (point-of-care human chorionic gonadotropin urine test). Male individuals were ineligible if they exclusively had sex with men.

Wave 1 participants provided names and contact information of sexual contacts. Trained staff notified contacts of their STI exposure and invited them to enroll. Sexual contacts were eligible if they were 16–40 years old, willing to undergo CT/NG screening, and willing to provide names and contact information of their sexual contacts from the prior 60 or 180 days. Pregnant female contacts and male contacts who exclusively had sex with men were eligible for waves 2–4; otherwise, exclusions were the same as for wave 1. Snowball recruitment of sexual contacts continued for up to four waves. All participants and sexual contacts who screened positive for CT/NG were treated free of charge, after sample collection, based on national guidelines (90). We constructed a sexual network diagram (Fig. S5). Sexual networks were small and isolated from each other, so we did not examine sexual network characteristics.

Demographic information and medical history were collected via in-person interview, and the participants completed social and behavioral history questionnaires (online or paper). Vaginal or penile urethral swabs were collected during reproductive tract examination, immediately stored at −18°C, and transferred daily on ice to the Institute for Genome Sciences at the University of Maryland School of Medicine (UMSOM), where they were stored at −80°C.

### Whole metagenome sequencing

Shotgun metagenomic data were generated for 138 samples (74 vaginal and 64 penile) as described previously (84). Swabs were resuspended in 1 mL of Amies transport medium. Genomic DNA was extracted using 200 µL of resuspended material and the MagAttract PowerMicrobiome DNA/RNA Kit (Qiagen) with bead-beating using TissueLyser II. Sequence libraries were generated from extracted DNA using the Illumina Nextera XT Flex kit. Procedures were performed according to manufacturer instructions and automated on the Hamilton STAR robotic platform. Libraries were sequenced on an Illumina NovaSeq 6000 (150 bp, paired-end) at Maryland Genomics at UMSOM. Host reads were identified and removed using BMtagger and the GRCh38 human genome as reference (91). Non-host reads were processed using fastp (v.0.21) (92). Metagenome taxonomic compositions were determined via mapping to VIRGO (59), and CSTs were assigned using VALENCIA (2). VALENCIA is a supervised classifier for the vaginal microbiome. The penile CST assignments we observed are consistent with prior unsupervised clustering results (25, 27–29), so we feel it is reasonable to use VALENCIA with penile samples in STING.

### Genome-resolved metagenomics

We *de novo* assembled each metagenome, individually, using metaSPAdes (60) with *k*-mer sizes of 21, 33, and 55. We divided assembled contigs into MAGs using MaxBin2 (v.2.2.7 -min_contig_length 500), MetaBAT2 (v.2.12.1, --maxEdges 500), and MetaDecoder (v.1.0.8, default settings) (61–63). We consolidated and refined bins using MetaWRAP (v.1.3.2, bin_refinement, default settings), which estimates bin quality using CheckM (v.1.1.3, default settings) (64, 65). We retained MAGs determined to be ≥90% complete and <5% contaminated for analyses. MAG taxonomy was assigned using GTDB-Tk (v.2.1.1 classify work_wf, database release 207) (66).

### Identification of strain concordance

We used inStrain to identify concordant strains between participants (68). We built inStrain profiles for each metagenome-MAG pair by mapping each metagenome’s reads against all retained MAGs using Bowtie2 (69). We summarized profiles using the inStrain compare function with Ward linkage, which reports coverage overlap and sequence similarity between each metagenome and each MAG. To measure sequence similarity, inStrain estimates popANI based on popSNPs, which are called when metagenomes share no alleles (major or minor) at a given site. To identify concordant strains, we applied stringent filters requiring ≥99.99% sequence similarity (popANI) over ≥50% shared coverage. We considered strains concordant between sexual contacts with <5 popSNP/Mbp to be sexually transmitted. Illumina NovaSeq 6000 error rates are ~0.1% (93); inStrain accounts for an error rate of 0.1%, so sequencing errors should not produce artifactual concordance/transmission events.

Several participant pairs appeared to be concordant for multiple strains of the same species. We assumed these participants were concordant for a single strain that mapped sufficiently well to multiple MAGs to meet concordance criteria when compared to multiple MAGs. For redundant events, we retained the event with the greatest number of base pairs compared and discarded remaining redundant events (215 events in 60 participant pairs discarded).

To identify and discard strain-concordance events likely resulting from contamination, we built inStrain profiles for the negative control sample. We used the same criteria as above to identify concordance between metagenomes and the negative control. We considered concordance to be due to contamination when two participants and the negative control were all concordant for the same strain, and we discarded four *G. leopoldii* concordance events likely resulting from contamination.

### Statistical analysis

We performed two differential abundance analyses using the ZicoSeq and ZicoSeq.plot functions of the GUniFrac package (v.1.7) in R (v.4.2.1, throughout) (67). We compared vaginal and penile metagenome relative abundances, and we compared penile metagenome relative abundances by CT status. ZicoSeq uses a permutation-based approach for multiple comparisons adjustment, and we controlled FWER at 1% using 999 permutations.

We constructed a network diagram depicting strain concordance events using the network function of the network package (v.1.18.0, throughout) (94, 95), as_tbl_graph, and activate functions of the tidygraph package (v.1.2.2) (96) and ggraph function of the ggraph package (v.2.1.0) (97) in R. We summarized network size using the network.edgecount and network.size functions of the network package in R.

We used Kruskal-Wallis tests to evaluate whether popSNP/Mbp between concordant strains differed across species or when strains were concordant between contacts vs non-contacts, whether vaginal and penile non-host reads differed, and whether per-MAG comparable sites differed between vaginal and penile metagenomes. We considered hypothesis tests significant at *P* < 0.05 (two-tailed, no multiple comparisons adjustment).

## Data Availability

Whole metagenome sequencing data are in the Short Read Archive (PRJNA798061). All scripts used to process and analyze metagenomes are available at GitHub. Additional data are available in the supplemental material.

## SUPPLEMENTAL MATERIAL

Figure S1 msphere.00030-24-s0001.tiff Differential abundance of taxa between penile metagenomes from male participants with CT/NG vs. without CT/NG.

Figure S2 msphere.00030-24-s0002.tiff Urogenital microbiota composition of female non-contacts with *Lactobacillus crispatus* and/or *Lactobacillus jensenii* concordance.

Figure S3 msphere.00030-24-s0003.tiff Urogenital microbiota composition of non-contacts with *Gardnerella swidsinskii* concordance.

Figure S4 msphere.00030-24-s0004.tiff Distribution of *Lactobacillus crispatus* relative abundances among female contacts in WSW dyads and male contacts in contact dyads.

Figure S5 msphere.00030-24-s0005.tiff STING sexual network diagram.

Figure S6 msphere.00030-24-s0006.tiff Penile urethral swabs yielded significantly fewer non-host reads than vaginal swabs.

Supplemental material legends msphere.00030-24-s0007.docx Supplemental figure and table legends.

Table S1 msphere.00030-24-s0008.xlsx MAG inventory.

Table S2 msphere.00030-24-s0009.xlsx STORMS checklist.

ASM does not own the copyrights to Supplemental Material that may be linked to, or accessed through, an article. The authors have granted ASM a non-exclusive, world-wide license to publish the Supplemental Material files. Please contact the corresponding author directly for reuse.
