# Supplementary figures and images for: Sexual transmission of urogenital bacteria: whole metagenome sequencing evidence from a sexual network study
Source: mSphere. 2024 Feb 15;9(3):e00030-24. doi: 10.1128/msphere.00030-24 (PMC10964427; doi:10.1128/msphere.00030-24)

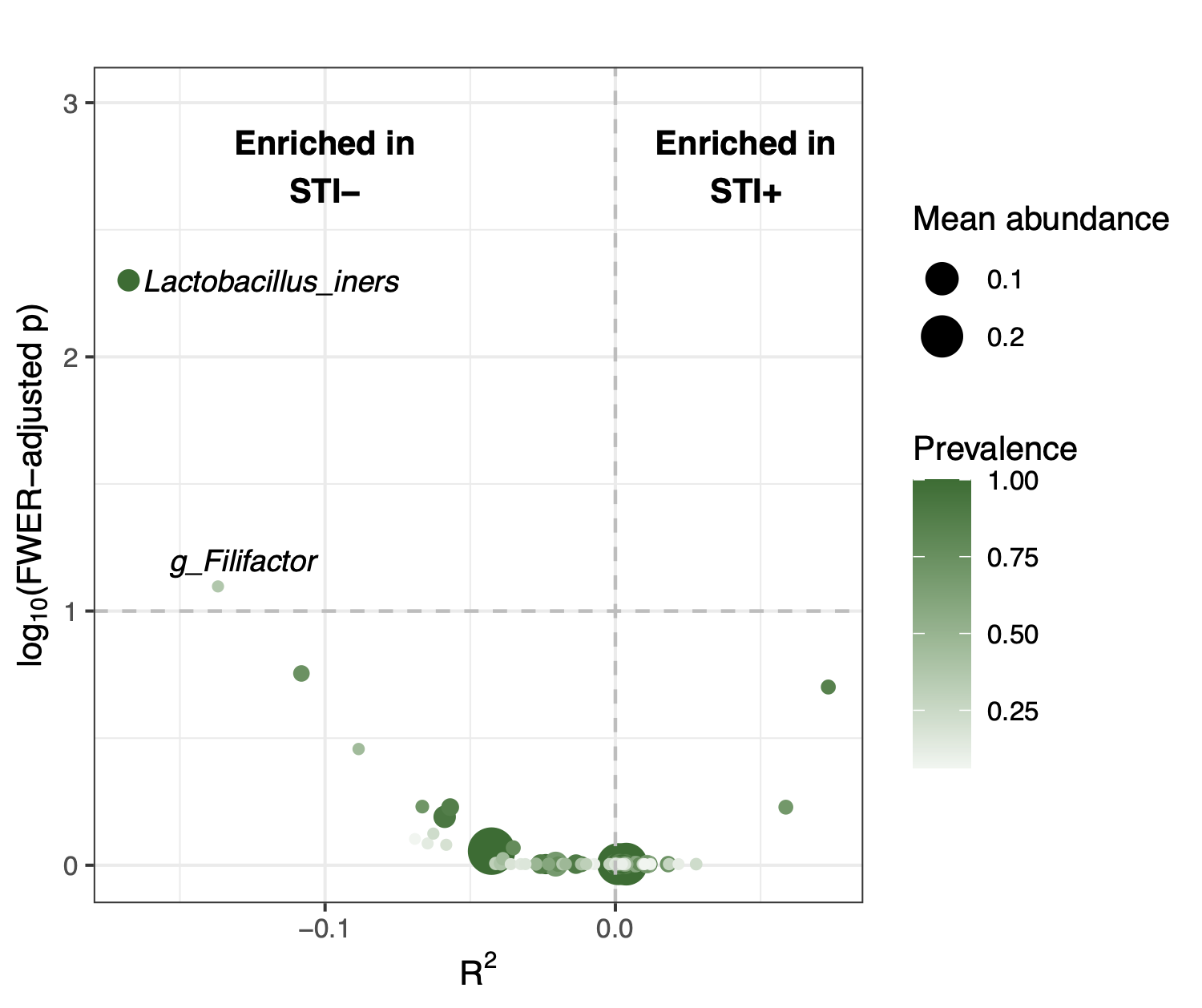

Supplement: Figure S1 — Differential abundance of taxa between penile metagenomes from male participants with CT/NG vs. without CT/NG. [file msphere.00030-24-s0001.tiff]

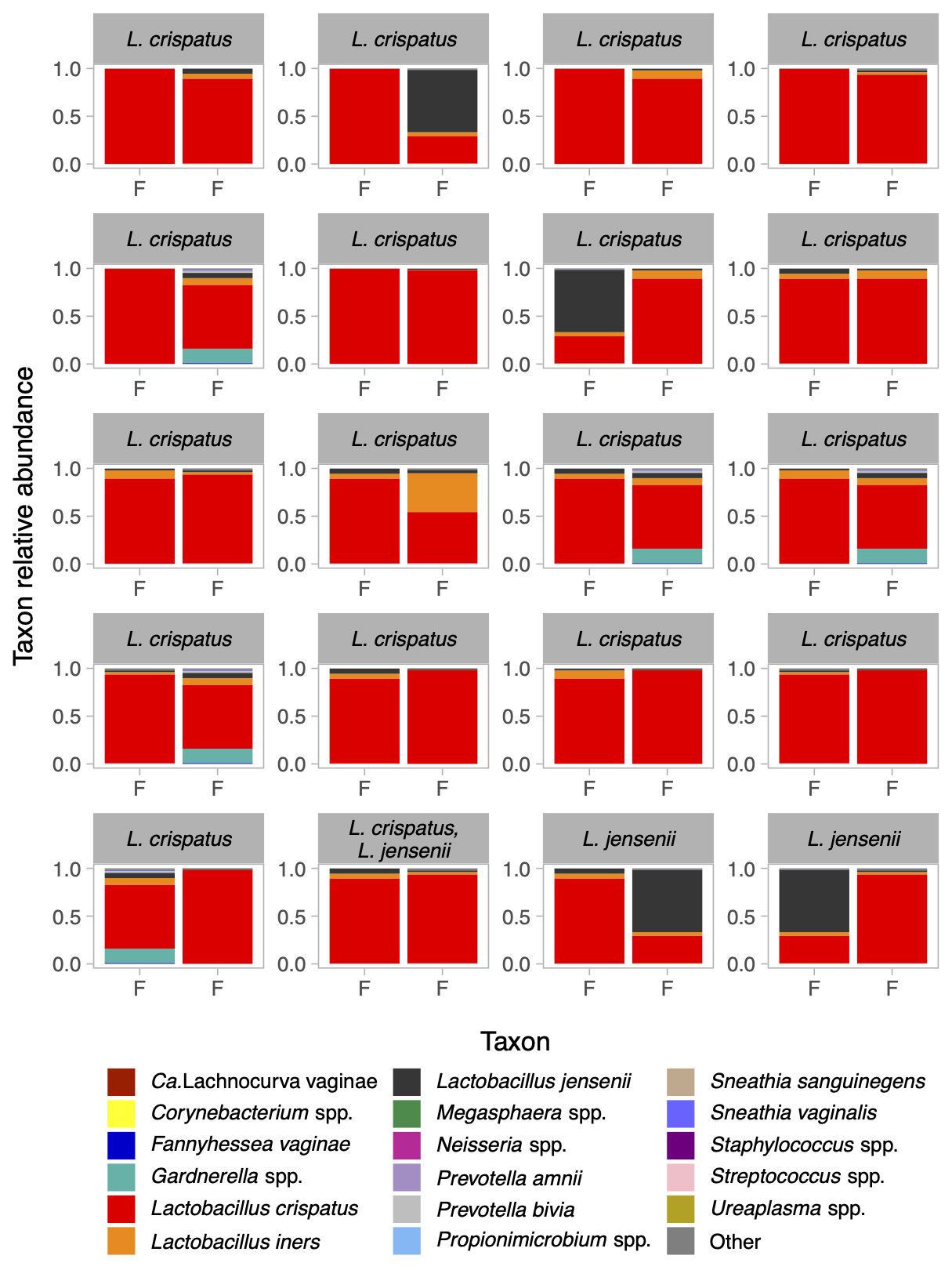

Supplement: Figure S2 — Urogenital microbiota composition of female non-contacts with Lactobacillus crispatus and/or Lactobacillus jensenii concordance. [file msphere.00030-24-s0002.tiff]

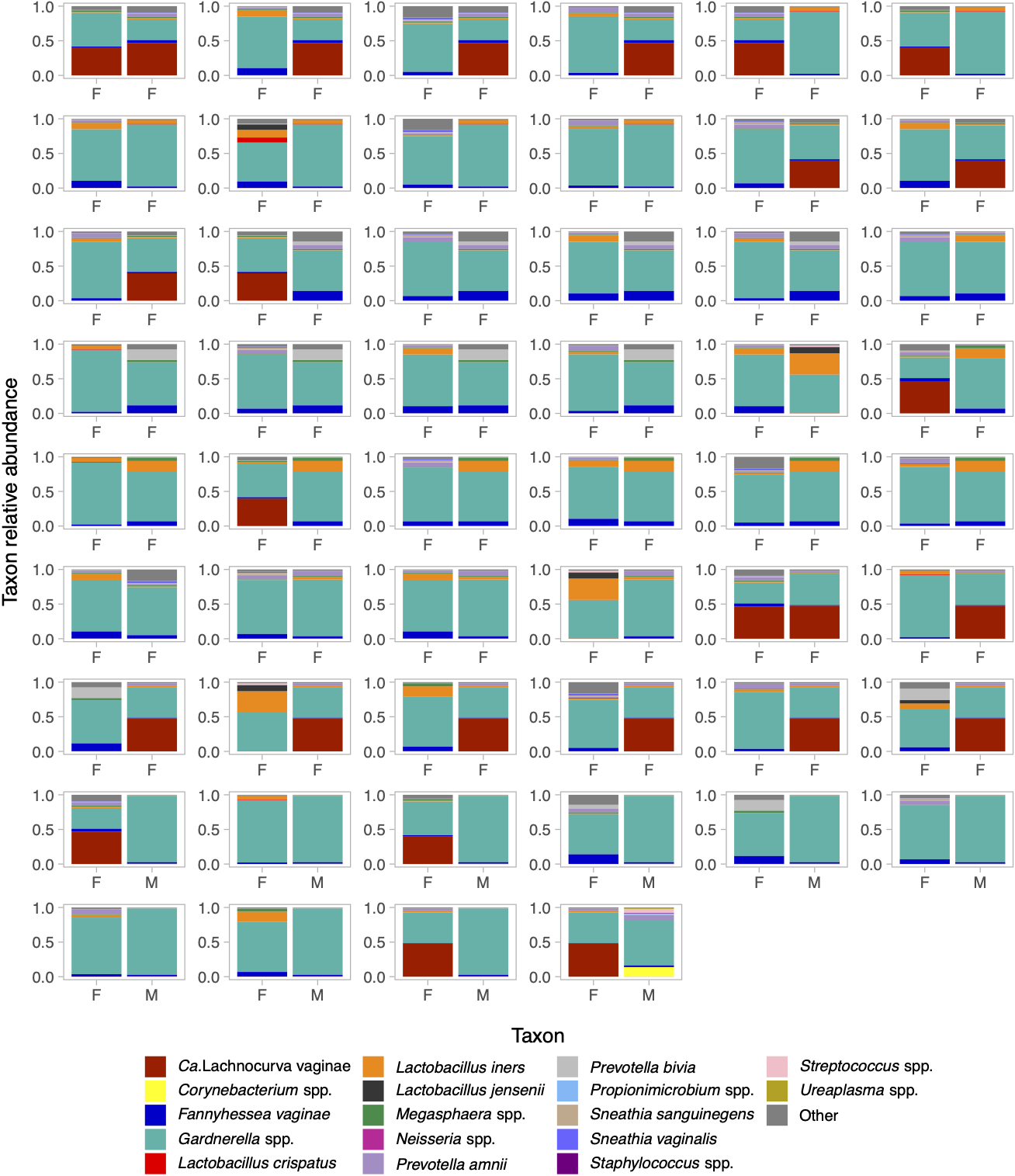

Supplement: Figure S3 — Urogenital microbiota composition of non-contacts with Gardnerella swidsinskii concordance. [file msphere.00030-24-s0003.tiff]

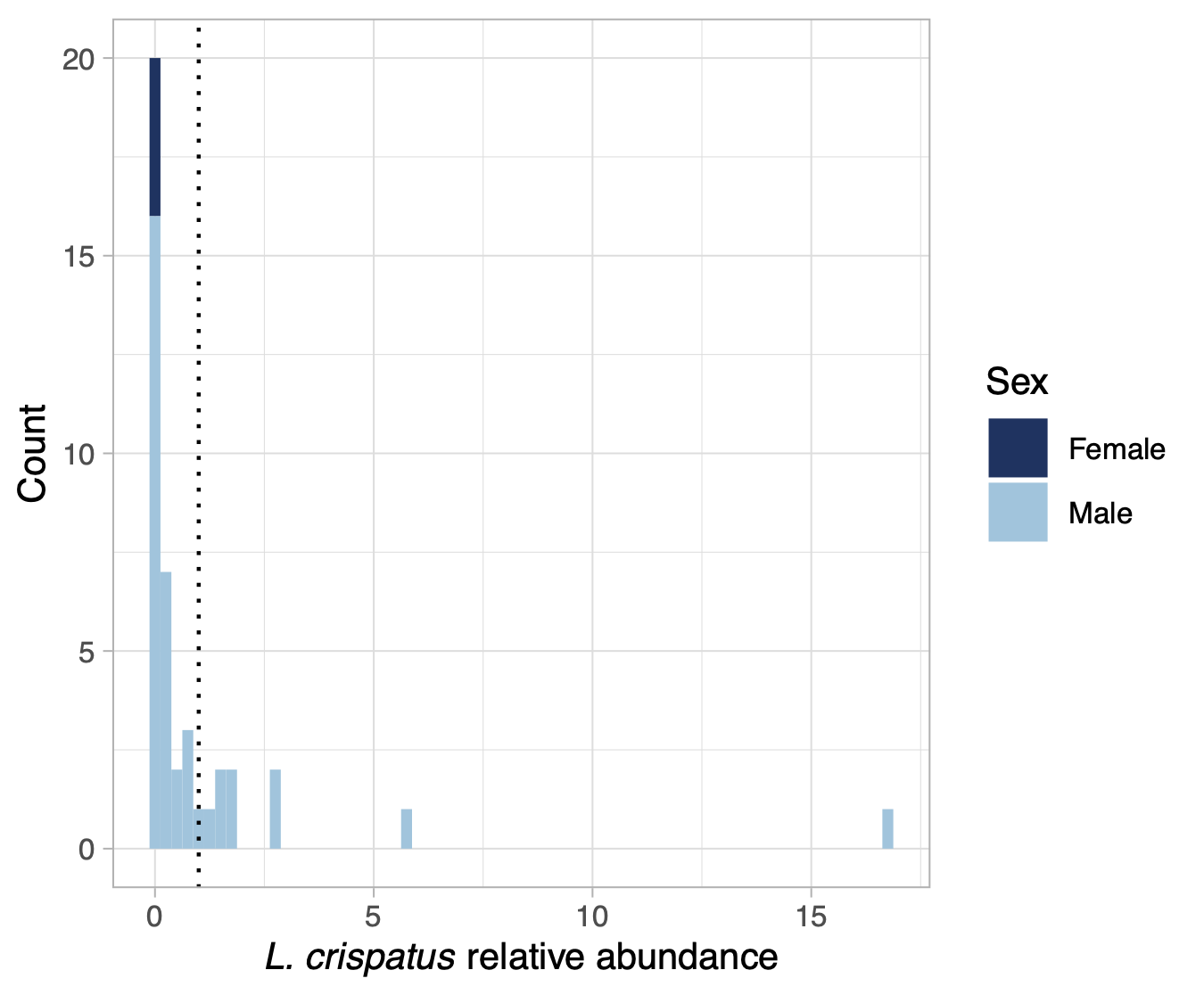

Supplement: Figure S4 — Distribution of Lactobacillus crispatus relative abundances among female contacts in WSW dyads and male contacts in contact dyads. [file msphere.00030-24-s0004.tiff]

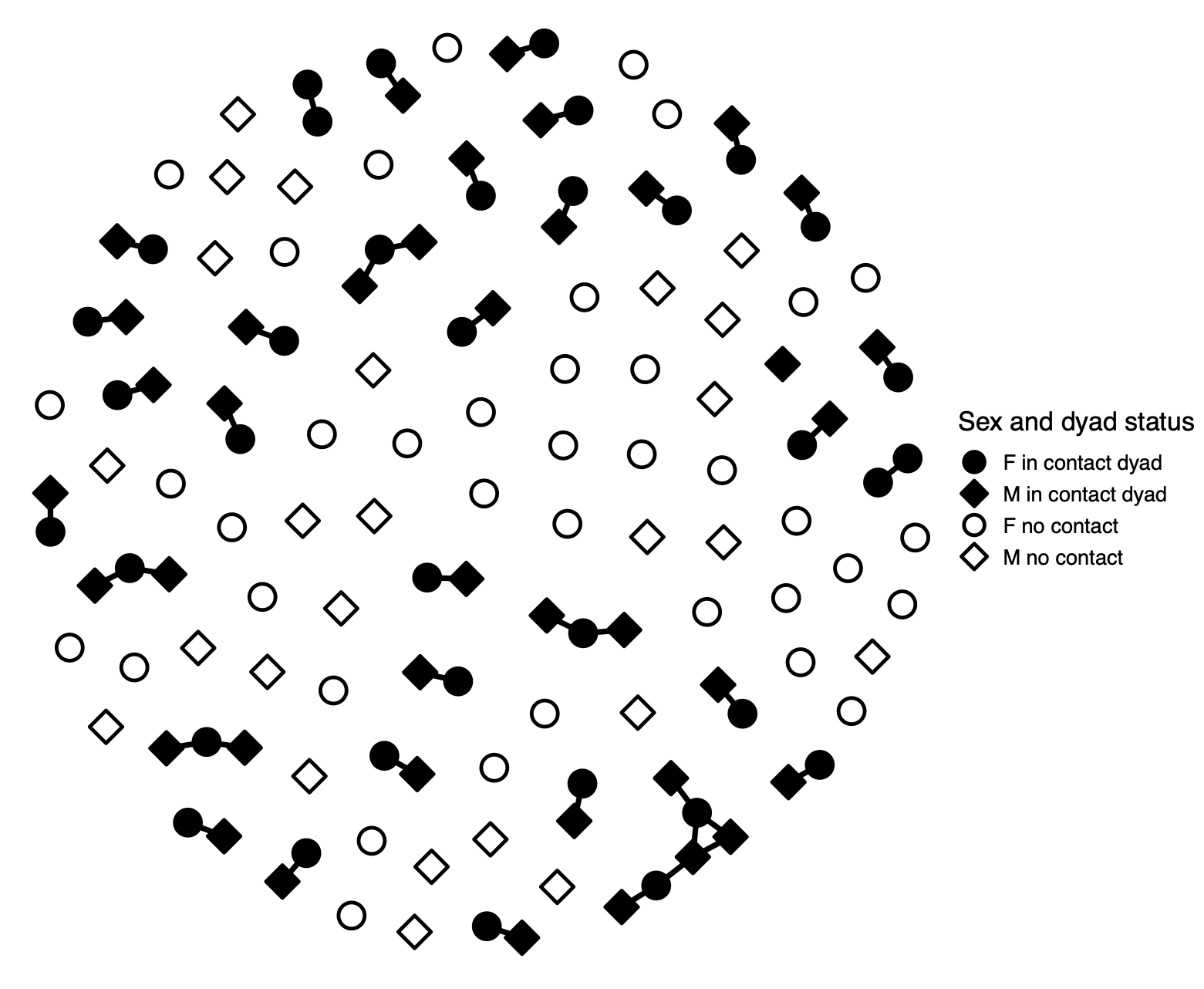

Supplement: Figure S5 — STING sexual network diagram. [file msphere.00030-24-s0005.tiff]

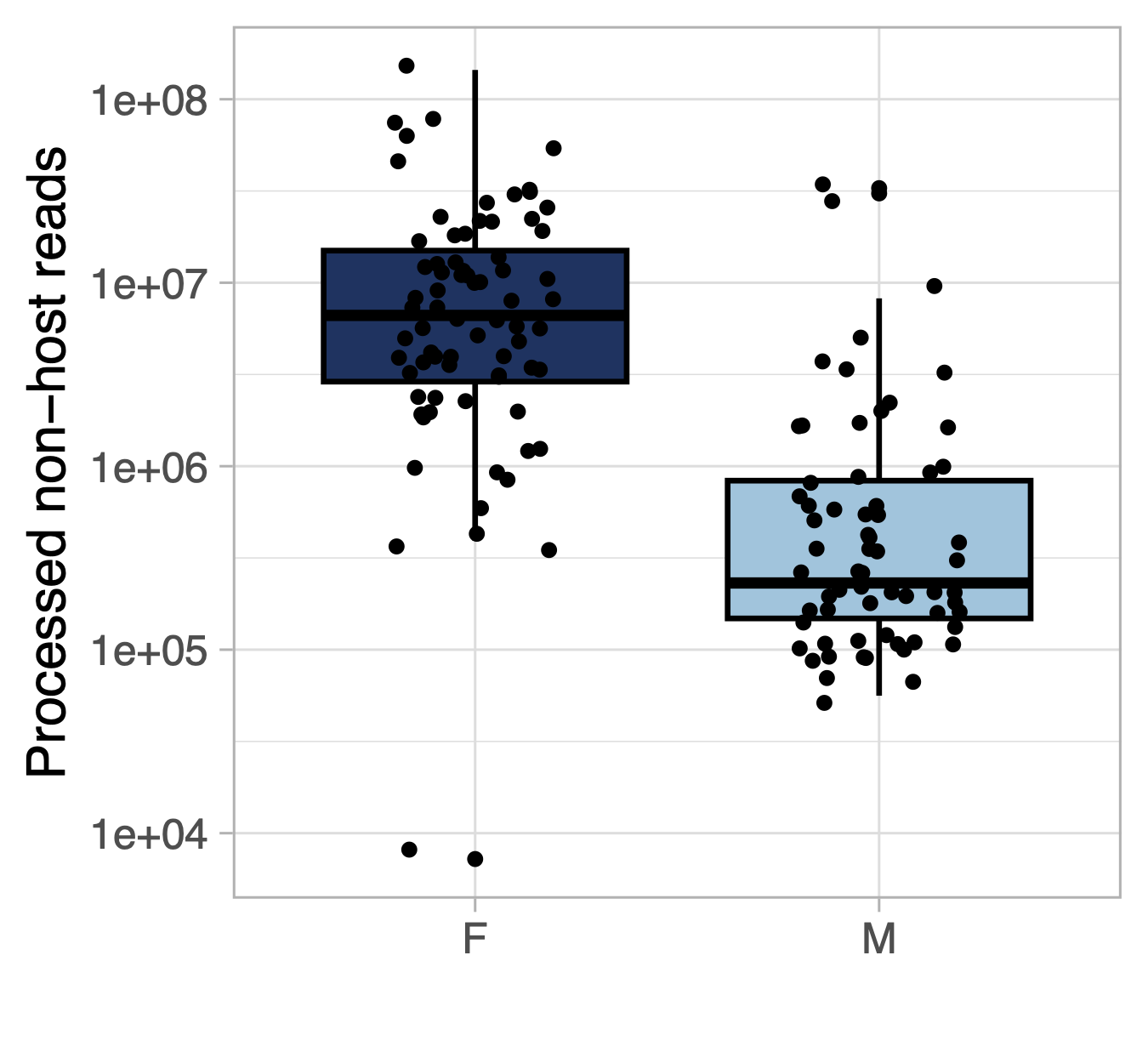

Supplement: Figure S6 — Penile urethral swabs yielded significantly fewer non-host reads than vaginal swabs. [file msphere.00030-24-s0006.tiff]
