# Supplementary material for: Sexual transmission of urogenital bacteria: whole metagenome sequencing evidence from a sexual network study
Source: mSphere. 2024 Feb 15;9(3):e00030-24. doi: 10.1128/msphere.00030-24 (PMC10964427; doi:10.1128/msphere.00030-24)
Supplement: Supplemental material legends — Supplemental figure and table legends. [file msphere.00030-24-s0007.docx]

**SUPPLEMENTAL MATERIAL**

**TABLE S1** STING MAG inventory. Includes metadata for each MAG included in analysis including: participant from whose metagenome the MAG was recovered, sex of the participant from whose metagenome the MAG was recovered, taxonomy, completeness, and contamination.

**FIG S1** Differential abundance of taxa between penile metagenomes from male participants with CT/NG vs. without CT/NG. Of 64 male participants, 2 are missing CT/NG data, so the sample size for this analysis is 62. R^2^ is the proportion of variance in metagenome composition (across penile samples) explained by a given taxon. The vertical dashed line at R^2^=0 indicates no difference in abundance between penile metagenomes with and without CT/NG, negative R^2^ values indicate enrichment in penile metagenomes without CT/NG. We controlled the FWER at 1%, as indicated by the horizontal dashed line at log_10_(FWER-adjusted p)=2. All taxa that are differentially abundant at this FWER fall above the horizontal line and are labeled.

**FIG S2** Urogenital microbiota composition of female non-contacts with *Lactobacillus crispatus* and/or *Lactobacillus jensenii* concordance. Each panel depicts urogenital microbiota composition for a unique dyad of non-contact female participants who had concordant *L. crispatus* and/or *L. jensenii* strains. The label at the top of each panel indicates the species of concordant strain(s). Participants that had concordance with >1 other participant are included multiple times, once for each concordance event.

**FIG S3** Urogenital microbiota composition of non-contacts with *Gardnerella swidsinskii* concordance. Each panel depicts urogenital microbiota composition for a unique dyad of non-contact participants who had a concordant *G. swidsinskii* strain. The x-axis labels indicate the sex of each participant. Participants that had concordance with >1 other participant are included multiple times, once for each concordance event.

**FIG S4** Distribution of *Lactobacillus crispatus* relative abundances among female contacts in WSW dyads and male contacts in contact dyads. The vertical dotted line is at 1% relative abundance.

**FIG S5** STING sexual network diagram. F, female; M, male.

**FIG S6** Penile urethral swabs yielded significantly fewer non-host reads than vaginal swabs. Kruskal-Wallis p=2x10^-16^. F, female; M, male.

**TABLE S2** STORMS reporting checklist.
